# The role of aberrant expression of T cell miRNAs affected by TNF-α in the immunopathogenesis of rheumatoid arthritis

**DOI:** 10.1186/s13075-017-1465-z

**Published:** 2017-12-01

**Authors:** Ning-Sheng Lai, Hui-Chun Yu, Chien-Hsueh Tung, Kuang-Yung Huang, Hsien-Bin Huang, Ming-Chi Lu

**Affiliations:** 1 Division of Allergy, Immunology and Rheumatology, Dalin Tzu Chi Hospital, Buddhist Tzu Chi Medical Foundation, No. 2, Minsheng Road, Dalin, Chiayi 62247 Taiwan; 2 School of Medicine, Tzu Chi University, Hualien City, Taiwan; 3 Department of Life Science and Institute of Molecular Biology, National Chung Cheng University, Minxiong, Chiayi Taiwan

**Keywords:** Rheumatoid arthritis, TNF-α, T cells, microRNAs, Apoptosis, JNK, ERK

## Abstract

**Background:**

Tumor necrosis factor-alpha (TNF-α) can cause diverse T cell dysfunctions in patients with rheumatoid arthritis (RA). It is involved in the regulation of microRNAs (miRNAs) expression in different cell types. We hypothesized that the expression of T cell miRNAs would be affected by TNF-α, and these miRNAs could participate in the immunopathogenesis of RA.

**Methods:**

Expression profiles of 270 human miRNAs in Jurkat cells, cultured in the presence or absence of TNF-α for 7 days were analyzed by real-time polymerase chain reaction. Potentially aberrantly expressed miRNAs were validated using T cell samples from 35 patients with RA and 15 controls. Transfection studies were conducted to search for gene expression and biological functions regulated by specific miRNAs.

**Results:**

Initial analysis revealed 12 miRNAs were significantly lower, whereas the expression level of miR-146a was significantly higher in Jurkat cells after being cultured with TNF-α for 7 days. Decreased expression of miR-139-3p, miR-204, miR-760, miR-524-5p, miR-136, miR-548d-3p, miR-214, miR-383, and miR-887 were noted in RA T cells. Expression levels of miR-139-3p, miR-204, miR-214, and miR-760 were correlated with the use of biologic agents. The transfection of miR-214 mimic suppressed TNF-α-mediated apoptosis of Jurkat cells. Increased phosphorylation of extracellular regulating kinase (ERK) and c-Jun N-terminal kinase (JNK) was noted in RA T cells and Jurkat cells after TNF-α exposure. Transfection of Jurkat cells with miR-214 mimic suppressed both the basal and TNF-α-mediated ERK and JNK phosphoryation.

**Conclusions:**

Among T cell miRNAs affected by TNF-α, the expression levels of nine miRNAs were decreased in T cells from patients with RA. The expression levels of miR-139-3p, miR-204, miR-214, and miR-760 increased in RA patients receiving biologic agents. The transfection of miR-214 reversed the TNF-α-mediated cells apoptosis and inhibited the phosphorylation of ERK and JNK in Jurkat cells.

**Electronic supplementary material:**

The online version of this article (doi:10.1186/s13075-017-1465-z) contains supplementary material, which is available to authorized users.

## Background

Rheumatoid arthritis (RA) is a common and disabling systemic autoimmune disease characterized by persistent joint inflammation. The presence of autoantibodies, immune complexes formation, abnormal T cell responses, T cell-independent cytokine networks, and aggressive tumor-like behavior of rheumatoid synovium are thought to be involved in the pathogenesis of RA [1]. Among these immunological dysfunctions, increased production of proinflammatory cytokines, especially tumor necrosis factor alpha (TNF-α) plays a critical role in the immunopathogenesis of RA [2]. TNF-α are known to regulate the immune system and to aggravate joint destruction in RA, and blockade of TNF-α could dramatically improve all clinical outcomes of RA [3, 4].

MicroRNAs (miRNAs) are small, non-coding RNA molecules of 21–24 base pairs that can control the expression of multiple gene targets at the post-transcriptional level. Abnormal expression of miRNAs, especially T cells from patients with RA was well documented. It could contribute to the pathogenesis of RA by facilitating the Th17 differentiation, inhibiting regulatory T cells differentiation, and causing an imbalance of the pro- and anti-inflammatory cytokine as well as an abnormal activation of T cells [5].

TNF-α is associated with diverse T cell dysfunctions in patients with RA [6, 7] and it also regulates the expression of miRNA in different cell types [8–10]. Li et al. showed that TNF-α upregulated miR-146a expression in RA T cells [11]. We believe that many more miRNAs affected by TNF-α will be found in T cells from RA patients and these miRNAs can participate in the immunopathogenesis of RA. We hypothesized that TNF-α-regulated miRNAs in T cells from RA patients could alter the expression of downstream target molecules and thereby contribute to the immunopathogenesis of RA.

## Methods

### Jurkat cells with chronic exposure to TNF-α

Purchased Jurkat cells (5 × 10^6^) (American Type Culture Collection, Manassas, VA, USA) were incubated in the presence or absence of TNF-α (20 ng/mL; Sigma-Aldrich, St. Louis, MO, USA) in Roswell Park Memorial Institute medium (RPMI)-1640 (Invitrogen, Carlsbad, CA, USA) containing heat-inactivated fetal bovine serum (10%), L-glutamine (2 mmol/L), penicillin (100 U/mL) and streptomycin (100 mg/mL) for 7 days.

### Assessment of miRNAs expression by real-time polymerase chain reaction (PCR)

Total RNA (including miRNAs) was extracted from purified T cells or Jurkat cells and the expression level of miRNAs was quantified as previously described [12].

### Isolation of T cells from patients with RA and controls

A total of 35 patients satisfying the 1987 American College of Rheumatology revised criteria for the classification of RA [13] were recruited, and 15 healthy individuals served as a control group. The study protocol was approved by the institutional review board of Buddhist Dalin Tzu Chi Hospital, Taiwan (No. B10503007). All participants signed informed consent prior to study participation. Blood samples were collected at least 12 h after the last dose of immunosuppressants to minimize their effects.

T cells were purified using anti-human CD3-coated magnetic beads (IMag Cell Separation System, BD Bioscience, Franklin Lakes, NJ, USA) according to the methods previously described [14]. The purity of T cells was checked using anti-human CD5 conjugated with fluorescein (Abcam, Cambridge, UK) and anti-human CD19 conjugated with phycoerythrin (Abcam).

The age (mean ± standard deviation) and sex ratio (female:male) were not significantly different between patients with RA (53.9 ± 11.4 years and 4.8:1) and controls (49.7 ± 9.4 years and 4:1). Among patients with RA, 24 (69%) were positive for rheumatoid factor and 31 (89%) were positive for anti-citrullinated protein antibodies (ACPAs). Serological data showed that the mean C-reactive protein (CRP) level was 0.64 ± 1.11 mg/dL and the mean erythrocyte sedimentation rate (ESR) was 16 ± 14 mm/h in patients with RA.

### Western blotting of cell lysates

Western blotting for the phosphorylation ratio of extracellular regulating kinase (ERK) and c-Jun N-terminal kinase (JNK) was performed as previously described [15]. Rabbit monoclonal antibodies against JNK, phospho-JNK (Thr183/Tyr185), ERK1/2, phospho-ERK1/2 (Thr202/Tyr204), and goat-anti-rabbit IgG conjugated with horseradish peroxidase (Cell Signaling Technology, Danvers, MA, USA) were used. Anti-β-actin antibody was used as internal control (Sigma-Aldrich).

### Transfection of miR-214 into Jurkat cells

Jurkat cells were transfected with miR-214 mimic, miR-214 inhibitor or scramble oligonucleotides (all from Ambion, Austin, TX, USA) using the conditions previously described [16], and then cultured with TNF-α (20 ng/mL) at 37 °C for 24 h or 48 h for further analysis of cell apoptosis or Western blot analysis, respectively.

### Detection of apoptosis by flow cytometry

Apoptotic rates were determined by doubly stained (FITC-annexin V and propidium iodide kit, BD Biosciences) Jurkat cells by flow cytometry (FACScan, Becton Dickinson, Franklin Lakes, NJ, USA) using Lysis II software.

### Statistical analysis

**Fig. 1:**
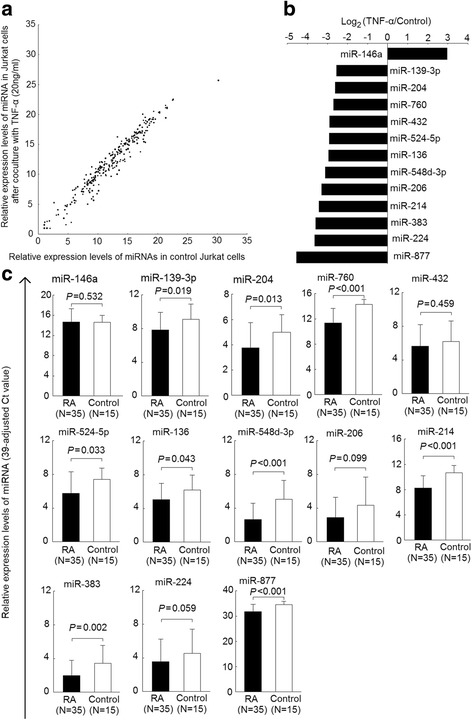
Altered expression of T cell miRNAs affected by TNF-α in patients with RA and healthy controls. **a** Expression profiles of 270 miRNAs in Jurkat cells cultured in the presence or absence of TNF-α (20 ng/mL) for 7 days, as determined by real-time PCR. Each scatter spot representing average normalized expression level of miRNA in three repeats of each treatment; (**b**) 13 miRNAs exhibiting aberrant expression in Jurkat cells cultured with TNF-α (20 ng/mL) for 7 days; (**c**) decreased expression of miR-139-3p, miR-204, miR-760, miR-524-5p, miR-136, miR-548d-3p, miR-214, miR-383, and miR-887 in RA T cells miRNA, compared with normal T cells. The relative expression level of miRNA was defined as (39 – Ct) after adjusting with an internal control (U6 small nuclear RNA)

**Fig. 2:**
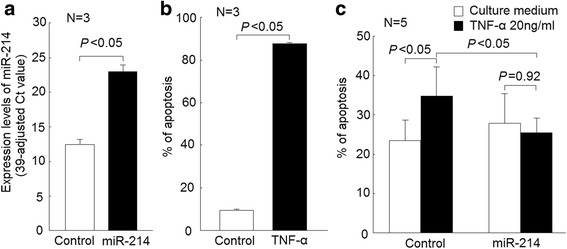
Effects of miR-214 mimic transfection in Jurkat cells apoptosis. **a** Remarkable elevation of miR-214 expression levels in Jurkat cells after transfection with miR-214 mimic versus controls (transfected with scramble oligonucleotides); (**b**) increased Jurkat cells apoptosis after cultured with TNF-α (20 ng/mL) for 7 days, compared with culture medium alone; (**c**) in Jurkat cells transfected scrambled oligonucleotides, the apoptotic rate of Jurkat cells was increased after cultured with TNF-α (20 ng/mL) for 24 h compared with those cultured with medium alone. The apoptotic rate was similar in Jurkat cells transfected with miR-214 mimic cultured either in the presence or absence of TNF-α (Fig. 2c)

**Fig. 3:**
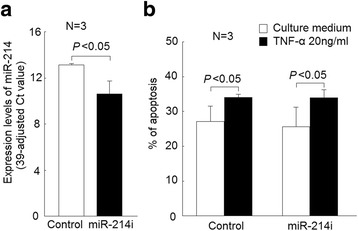
Effects of miR-214 inhibitor (miR-214i) transfection in Jurkat cells apoptosis. **a** Decreased miR-214 expression in Jurkat cells after transfection with miR-214 inhibitor versus scramble oligonucleotides; (**b**) in Jurkat cells transfected miR-214 inhibitor or controls, the apoptotic rate was increased after cultured with TNF-α (20 ng/mL) for 24 h compared with those cultured with medium alone. Whether cultured with TNF-α or not, the apoptotic rate of Jurkat cells was not different between those transfected with miR-214 inhibitors and the controls

**Fig. 4:**
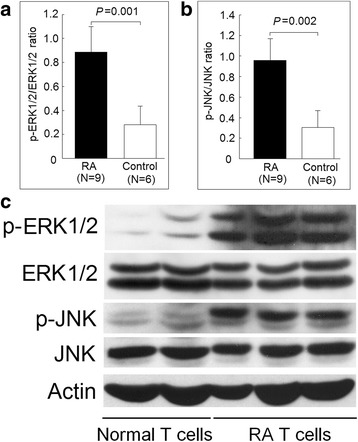
Comparison of ERK and JNK protein phosphorylation in T-cell lysates from RA and control groups as detected by Western blot analysis. Increased (**a**) ERK and (**b**) JNK phosphorylation in nine patients with RA and six healthy controls, normalized to actin expression; (**c**) ERK and JNK protein phosphorylation in T cell lysates of three patients with RA and two healthy controls as representative tests

**Fig. 5:**
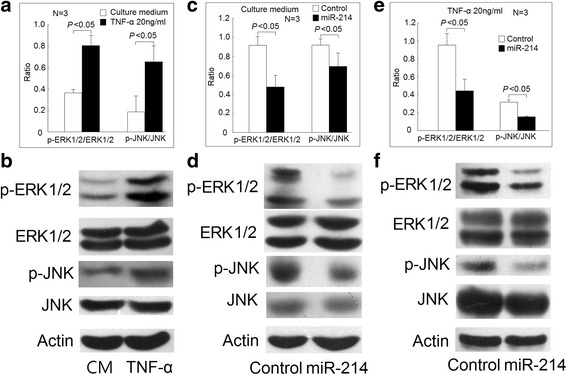
Effect of miR-214 on ERK and JNK protein phosphorylation in Jurkat cells. **a** The phosphorylation ratio of ERK and JNK increased in Jurkat cells after being cultured with TNF-α (20 ng/mL) for 48 h compared with those cultured with medium (CM) alone and (**b**) a representative case. **c** In Jurkat cells after transfection with miR-214 mimic or scramble oligonucleotides cultured with medium alone for 48 h, the phosphorylation ratio of ERK and JNK decreased in those transfected with miR-214 mimic compared with the control groups and (**d**) a representative case. **e** In Jurkat cells after transfection with miR-214 mimic or scramble oligonucleotides cultured with TNF-α (20 ng/mL) for 48 h, the phosphorylation ratio of ERK and JNK decreased in those transfected with miR-214 mimic compared with the control groups and (**f**) a representative case

Simple and multiple linear regression analyses were conducted to obtain correlation coefficients and assessing statistical significance for various parameters in Tables 1 and 2, respectively. All comparisons in Figs 1, 2, 3, 4 and 5 were assessed using Mann-Whitney *U* test. Statistical significance was set at *p* < 0.05. All statistical analyses were performed using Stata statistical software (StataCorp, College Station, TX, USA).

**Table 1:** Associations between miRNAs expression levels in T cells and clinical parameters in rheumatoid arthritis patients

miRNA	Age (per 10 years	Sex (male/female)	CRP (mg/dL)	ACPAs (per 10 IU/mL)	Positivity of RF (yes/no)	Sulfalsalazine usage (yes/no)	MTX dosage (mg/week)	Leflunomide usage (yes/no)	Steroid dosage equivalent to perdnisolone (mg/day)	Biologic agent^a^ usage (yes/no)
miR-139-3p	0.07 (0.816)	–0.35 (0.732)	–0.26 (0.460)	-0.03 (0.373)	**–1.73 (0.031)**	–0.50 (0.683)	0.06 (0.581)	0.51 (0.508)	0.50 (0.054)	**2.02 (0.007)**
miR-204	0.15 (0.615)	–1.36 (0.128)	–0.33 (0.285)	-0.04 (0.152)	–0.63 (0.385)	–1.27 (0.233)	–0.06 (0.484)	0.26 (0.700)	0.09 (0.707)	**2.04 (0.002)**
miR-214	–0.26 (0.344)	–0.05 (0.949)	**–0.72 (0.010)**	-0.01 (0.515)	–0.96 (0.158)	–1.56 (0.116)	–0.10 (0.287)	1.02 (0.107)	0.03 (0.900)	**1.50 (0.016)**
miR-383	–0.03 (0.891)	–0.82 (0.309)	0.11 (0.687)	0.02 (0.497)	–0.12 (0.854)	**–2.72 (0.003)**	**–0.22 (0.009)**	–0.26 (0.672)	**–0.53 (0.009)**	0.41 (0.507)
miR-524-5p	–0.39 (0.307)	–0.93 (0.435)	–0.41 (0.313)	0.00 (0.921)	–1.07 (0.266)	–1.32 (0.353)	–0.07 (0.568)	0.69 (0.447)	0.09 (0.775)	0.63 (0.488)
miR-548d-3p	–0.19 (0.494)	–0.14 (0.870)	–0.45 (0.123)	0.05 (0.051)	0.14 (0.845)	–1.66 (0.098)	–0.01 (0.880)	–0.43 (0.505)	0.02 (0.926)	0.42 (0.524)
miR-760	–0.40 (0.253)	0.29 (0.795)	–0.23 (0.553)	-0.01 (0.780)	–1.08 (0.226)	–1.56 (0.234)	–0.08 (0.485)	**1.73 (0.035)**	0.51 (0.074)	**2.32 (0.004)**
miR-877	–0.48 (0.235)	–0.27 (0.833)	–0.04 (0.922)	-0.02 (0.554)	0.05 (0.965)	–0.32 (0.830)	–0.08 (0.563)	1.28 (0.175)	0.36 (0.266)	1.30 (0.174)

Values shown are correlation coefficients and (p values) from simple linear regression, and those in bold represent p < 0.05

*ACPAs* anti-citrullinated protein antibodies, *CRP* C-reactive protein, *miRNA* microRNAs, *MTX* methotrexate, *RF* rheumatoid factor

^a^Biologic agent including: tumor necrosis factor antagonists, abatacept, and tocilizumab

Values shown are correlation coefficients and (*p* values) from simple linear regression, and those in bold represent *p* < 0.05

**Table 2:** Adjusted associations between miRNAs expression levels in T cells and parameters in rheumatoid arthritis patients

miRNA	Age (per 10 years	Sex (male/female)	CRP (mg/dL)	ACPAs (per 10 IU/mL)	Positivity of RF (yes/no)	Sulfasalazine usage (yes/no)	MTX dosage (mg/week)	Leflunomide usage (yes/no)	Steroid dosage equivalent to perdnisolone (mg/day)	Biologic agent^a^ usage (yes/no)
miR-139-3p	1.03 (0.68–1.56)	0.78 (0.21–2.84)	–	–	**0.33 (0.12**–**0.94)**	–	–	–	1.37 (0.98–1.92)	**2.84 (1.06**–**7.64)**
miR-204	0.83 (0.59–1.16)	**0.27 (0.09**–**0.78)**	–	–	–	–	–	–	–	**4.48 (2.02**–**9.90)**
miR-214	0.88 (0.62–1.24)	0.60 (0.20–1.80)	**0.65 (0.45**–**0.94)**	–	–	–	–	–	–	**2.44 (1.08**–**5.48)**
miR-383	1.08 (0.77–1.52)	0.71 (0.24–2.09)	–	–	–	0.33 (0.08–1.36)	0.93 (0.82–1.05)	–	0.81 (0.60–1.10)	–
miR-548d-3p	0.80 (0.52–1.22)	0.55 (0.14–2.16)	–	0.62 (0.13–2.96)	–	–	–	–	–	–
miR-760	0.72 (0.46–1.11)	0.97 (0.25–3.81)	–	–	–	–	–	2.54 (0.94–6.90)	1.35 (0.95–1.93)	**3.66 (1.31**–**10.22)**

Values shown are fold change (95% confidence interval) calculated using multiple linear regression analysis, and those in bold represent p < 0.05

*ACPAs* anti-citrullinated protein antibodies, *CRP* C-reactive protein, *miRNA* microRNAs, *MTX* methotrexate, *RF* rheumatoid factor

^a^Biologic agent including: tumor necrosis factor antagonists, abatacept, and tocilizumab

## Results

### Identification of the chronic TNF-α exposure affected expression of miRNAs in Jurkat cells

Expression profiles of 270 miRNAs in Jurkat cells cultured in the presence or absence of TNF-α (20 ng/mL) for 7 days are displayed in Fig. 1a, with each scatter spot represents the average of three adjusted miRNA levels from each group. The expression levels of 12 miRNAs, including miR-139-3p, miR-204, miR-760, miR-432, miR-524-5p, miR-136, miR-548d-3p, miR-206, miR-214, miR-383, miR-224, and miR-887 were significantly lower, whereas the expression level of miR-146a was significantly higher, in Jurkat cells after being cultured with TNF-α for 7 days (fold change > 4, *p* < 0.05, Fig. 1b).

### Expression profiles of T cell miRNAs affected by TNF-α miRNAs from patients with RA and healthy controls

The purities of T cells were all greater than 98.75%, and a representative example was shown in Additional file 1: Figure S1. The expression levels of the T cell miRNAs affected by TNF-α were investigated in T cells from patients with RA and healthy controls. The expression of miR-139-3p, miR-204, miR-760, miR-524-5p, miR-136, miR-548d-3p, miR-214, miR-383, and miR-887 was found to be significantly lower in RA T cells (*p* < 0.05), compared with controls (Fig. 1c). The fold changes of expression levels for these miRNAs were 0.42-fold for miR-139-3p, 0.43-fold for miR-204, 0.13-fold for miR-760, 0.32-fold for miR-524-5p, 0.45-fold for miR-136, 0.19-fold for miR-548d-3p, 0.37-fold for miR-214;0.36-fold for miR-383, and 0.14-fold for miR-887, compared with controls. After adjusting for age and sex, the expression levels of these nine TNF-α-regulated miRNAs remained significantly lower in T cells from patients with RA compared with the healthy controls.

### Correlations of miRNAs expression levels and clinical parameters in patients with RA

The relationships between various clinical parameters and the expression levels of miRNAs in RA T cells were investigated through simple (Table 1) and multiple linear regression analyses (Table 2). With simple linear regression analysis, expression levels of miR-139-3p showed a significant correlation with rheumatoid factor (RF) positivity and the use of biologic agents. The expression levels of miR-204 showed a significant correlation with the use of biologic agents. The expression levels of miR-214 showed a significant correlation with the serum CRP levels and the use of biologic agents. The expression levels of miR-383 showed a significant correlation with the use of sulfalsalazine, methotrexate, and daily steroid dosage. The expression levels of miR-760 showed a significant correlation with the use of leflunomide and biologic agents.

After adjusting for age and sex using multiple linear regression analysis (Table 2), RA patients with RF positivity had a significant 0.33-fold decrease (*p* = 0.039; 95% confidence interval [CI] 0.12–0.94) and the use of biologic agents had a significant 2.84-fold increase (*p* = 0.039; 95% CI 1.06–7.64) in miR-139-3p expression levels. RA patients of male sex had a significant 0.27-fold decrease (*p* = 0.019; 95% CI 0.09–0.78) and the use of biologic agents had a significant 4.48-fold increase (*p* = 0.001; 95% CI 2.02–9.90) in miR-204 expression levels. Moreover, RA patients with each 1 mg/dL increment of CRP levels had a significant 0.65-fold decrease (*p* = 0.025; 95% CI 0.45–0.94) and the use of biologic agents had a significant 2.44-fold increase (*p* = 0.032; 95% CI 1.08–5.48) in miR-214 expression levels. Furthermore, the use of biologic agents in RA patients had a significant 3.66-fold increase (*p* = 0.015; 95% CI 1.31–10.22) in miR-760 expression levels.

### Transfection of miR-214 mimic suppressed TNF-α-mediated apoptosis of Jurkat cells

Since the expression levels of miR-214 were statistically significant correlated with the serum CRP levels and the use of biologic agents. We further surveyed the functional effects of miR-214 in T cells. First, we successfully transfected miR-214 mimic into Jurkat cells (Fig. 2a). It is known that TNF-α could enhance Jurkat cells apoptosis. In Jurkat cells cultured with TNF-α for more than 7 days, a high percentage of Jurkat cells became apoptotic (Fig. 2b). Then, we transfected miR-214 mimic and controls into Jurkat cells and cultured them in the presence or absence of TNF-α for 24 hours. The apoptotic rate of Jurkat cells transfected with scrambled oligonucleotides increased after cultured with TNF-α (20 ng/mL) for 24 h compared with those cultured with medium alone. In contrast, the apoptotic rate was similar in Jurkat cells transfected with miR-214 mimic after cultured in the presence or absence of TNF-α (Fig. 2c).

### Transfection of miR-214 inhibitor did not affect survival of Jurkat cells

First, we successfully transfected miR-214 inhibitor into Jurkat cells and the expression levels of miR-214 were modest but significantly lower in Jurkat cells transfected with miR-214 inhibitor compared with the controls (Fig. 3a). Then, we transfected miR-214 inhibitor and controls into Jurkat cells and cultured in the presence or absence of TNF-α for 24 hours. The apoptotic rates of Jurkat cells were both significantly elevated in Jurkat cells transfected with miR-214 inhibitor and controls. Whether cultured in the presence or absence of TNF-α, there were no significant differences in the apoptotic rates of Jurkat cells transfected with miR-214 inhibitor compared with the controls (Fig. 3b).

### Increased phosphorylation of JNK and ERK in T cells from patients with RA

TNF-α is known to trigger the activation of mitogen-activated protein kinases (MAPKs). Increased phosphorylation of ERK and JNK has been documented in T cells from patients with RA in previous research [17, 18]. Therefore, we evaluated and confirmed that the phosphorylation ratio of ERK and JNK was increased in T cells from patients with RA (Fig. 4). Next, we demonstrated that TNF-α could increase the phosphorylation ratio of ERK and JNK in Jurkat cells (Fig. 5a and b). It has been reported that miR-214 could inhibit the protein expression of Ras [19], an upstream activator of ERK and JNK. We speculated that the decreased expression of miR-214 might contribute to the activation of ERK and JNK. First, we transfected Jurkat cells with miR-214 mimic or scrambled oligonucleotides and cultured with culture medium for 48 h. We found that the phosphorylation ratio of ERK and JNK decreased in Jurkat cells transfected with miR-214 mimic compared with those transfected with scrambled oligonucleotides (Fig. 4c and d). Then, we transfected Jurkat cells with miR-214 mimic or scrambled oligonucleotides and then cultured with TNF-α (20 ng/mL) for 48 h. We found that the phosphorylation ratio of ERK and JNK also decreased in Jurkat cells transfected with miR-214 mimic compared with those transfected with scrambled oligonucleotides (Fig. 4e and f).

## Discussion

Our study showed that being chronically exposed to TNF-α in Jurkat cells affected the expression of 13 miRNAs, and nine miRNAs were found to be downregulated in T cells from RA patients. Furthermore, four miRNAs expression levels were upregulated after the use of biologic agents. These results supported that TNF-α can play a critical role in the immunopathogenesis of RA. The expression of several miRNAs was closely related to the use of biologic agents. Their functional roles need further investigation. The TNF-α is a pleiotropic cytokine that plays many roles in the pathogenesis of RA, such as regulating cell proliferation/apoptosis, balancing cytokines, and promoting inflammation reactions [20]. Elevated serum concentration of TNF-α in patients with RA was well documented decades ago [21]. In T cells, short-term TNF-α exposure is important for inflammatory response, but sustained TNF-α expression, such as in patients with RA can have a different impact on T cell function. The characteristics of T cells chronically exposed to TNF-α can regulate gene expression, such as overexpression of CD69 and underexpression of CD28; promote nondeletional proliferative hyporesponsiveness; suppress cytokine production, and alter T cell receptor signal transduction [7].

Several studies have investigated the effect of TNF-α in miRNAs expression among neurons, muscle, and endothelium cells [8–10]. Among them, decreased expression of miR-206 was noted in myogenic cells, and can potentially affect MAPK pathways [9]. Decreased expression of miR-206 in Jurkat cells after chronic exposure to TNF-α was also noted in our study. However, different cell types and duration of TNF-α exposure might have affected the study results. Li et al. showed that the expression level of miR-146a was increased in CD4+ T cells of RA patients and was closely correlated with TNF-α level [11]. Our results also showed that the expression of miR-146a increased in Jurkat cells after being cultured with TNF-α, but not in RA T cells. We noted that the disease activities were high in patients with RA in Li et al. [11] (mean ESR = 68 mm/h), whereas the disease activities in our RA patients were lower (mean ESR = 16 mm/h). In addition, different ethnic groups and treatments might have affected the results.

Initially, our studies showed that among the expression of T cell miRNAs affected by TNF-α in Jurkat cells, the expression levels of miR-139-3p, miR-204, miR-760, miR-383, miR-524-5p, miR-136, miR-548d-3p, and miR-214 were significantly decreased in RA T cells. Since the expression levels of miR-214 were significantly correlated with the serum CRP levels and the use of biologic agents, we further investigated the functional effects of miR-214 in T cells. After the ligation of TNF-α with its receptors, several downstream molecules were recruited and activated, leading to the phosphorylation of ERK and JNK [6]. We found that miR-214 could suppress both the basal level and TNF-α-induced ERK and JNK phosphorylation and apoptosis in Jurkat cells. The phosphorylation of JNK is a strong apoptosis inducer [22] and the inhibition of JNK phosphorylation can lead to decreased Jurkat cells apoptosis. In contrast, the activation of ERK is a survival signal for T cells. However, strong and transient activation of ERK can also lead to T cell death [23–25]. The pathologic function of miRNA-214 has been extensive investigated for different cancers, and its effects in carcinogenesis are complex and diverse [26]. The impact of dysregulated miR-214 for cell apoptosis is still controversial [27–30].

As for the signaling pathway, the miR-214 not only could suppress the upstream signaling molecule RAS in MAPK pathways [17], it might directly suppress the expression of JNK and ERK [31, 32]. Among various systemic autoimmune diseases, decreased expression of miR-214 was found in patients with multiple sclerosis [33]. It has been found that increased expression of miR-214 in T cells upon activation [34] and increased miR-214 expression could promote the release of inflammatory cytokines, such as TNF-α and interleukin (IL)-6 in murine macrophages [35]. Therefore, the pathologic role of decreased expression of miR-214 in T cells from patients with RA might be an insufficient negative feedback to stop the inflammatory response similar to the role of miR-146a in the immunopathogenesis of RA [5].

Among other miRNAs affected by TNF-α in RA T cells, miR-204 was found to be downregulated in human retinal pigment epithelial cells after exposure to a mixture of inflammatory cytokines containing interferon gamma, TNF-α, and IL-1 beta [36]. In addition, it is of interest to note that the expression level of miR-524-5p was increased in T cells from patients with systemic lupus erythematosus [37], but decreased in those from patients with RA. A differential expression pattern between systemic lupus erythematosus and RA T cells was found in the expression of miR-146a and miR-21 [5]. It has been reported that the expression of miR-146a, miR-155, and miR-132 is upregulated in monocytes after exposure with TNF-α [38], but our study showed only the expression miR-146a was increased in T cells after chronic exposure to TNF-α. We believe that it might be related to the different cell lines used.

Among the nine T-cell miRNAs affected by TNF-α and downregulated in RA T cells, the expression levels of miR-139-3p, miR-204, miR-214, and miR-760 were increased in patients using biologic agents. The majority of biologic agents used were anti-TNF agents, including etanercept and adalimumab. Only one patient received abatacept and three patients received tocilizumab. Previous studies have shown that abatacept could potently suppress activated T cell-mediated TNF-α secretion from macrophages [39]. Moreover, tocilizumab could suppress the differentiation of Th17 cells by blocking IL-6 signaling and thus inhibit the IL-17-mediated TNF-α production in innate immunity [40]. In addition, we did not find any differences in the miRNA expression patterns in the eight TNF-regulated miRNAs between anti-TNF or non-TNF biologic agents.

## Conclusions

Our study showed that TNF-α affected the expression of miRNAs in Jurkat cells. Among these miRNAs, the expression levels of nine miRNAs were decreased in T cells from patients with RA. The expression levels of miR-139-3p, miR-204, miR-214, and miR-760 increased in RA patients using biologic agents. The transfection of miR-214 mimic reversed the TNF-α mediated cell apoptosis as well as ERK and JNK phosphorylation and thus appeared to be involved in the immunopathogenesis of RA.

## Additional file

Additional file 1: Figure S1. A representative flow cytometry analysis of T cell purity. Purity of T cells was assessed by staining with anti-human CD5 conjugated with fluorescein (FITC) and without anti-human CD19 conjugated with phycoerythrin (PE). The purity of T cells was 99.5% in this sample from a patient with rheumatoid arthritis. (DOC 63 kb)

## Abbreviations

ACPAs: Anti-citrullinated protein antibodies
CRP: C-reactive protein
ERK: Extracellular regulating kinase
ESR: Erythrocyte sedimentation rate
IL: interleukin
JNK: c-Jun N-terminal kinase
MAPKs: Mitogen-activated protein kinases
miRNAs: MicroRNAs
MTX: Methotrexate
PCR: polymerase chain reaction
RA: Rheumatoid arthritis
RF: Rheumatoid factor
RPMI: Roswell Park Memorial Institute medium
TNF-α: Tumor necrosis factor alpha
