# Supplementary figures and images for: The role of aberrant expression of T cell miRNAs affected by TNF-α in the immunopathogenesis of rheumatoid arthritis
Source: Arthritis Res Ther. 2017 Dec 1;19:261. doi: 10.1186/s13075-017-1465-z (PMC5709919; doi:10.1186/s13075-017-1465-z)

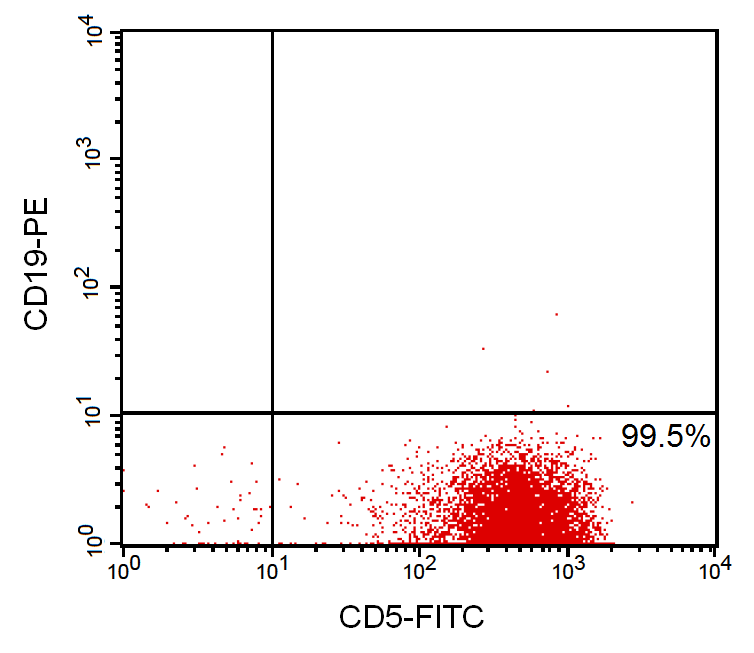

Supplement: Additional file 1: Figure S1. — A representative flow cytometry analysis of T cell purity. Purity of T cells was assessed by staining with anti-human CD5 conjugated with fluorescein (FITC) and without anti-human CD19 conjugated with phycoerythrin (PE). The purity of T cells was 99.5% in this sample from a patient with rheumatoid arthritis. (DOC 63 kb) [file 13075_2017_1465_MOESM1_ESM.doc]
